# If I Were You: Perceptual Illusion of Body Swapping

**DOI:** 10.1371/journal.pone.0003832

**Published:** 2008-12-03

**Authors:** Valeria I. Petkova, H. Henrik Ehrsson

**Affiliations:** Department of Neuroscience, Karolinska Institutet, Stockholm, Sweden; University of Sydney, Australia

## Abstract

The concept of an individual swapping his or her body with that of another person has captured the imagination of writers and artists for decades. Although this topic has not been the subject of investigation in science, it exemplifies the fundamental question of why we have an ongoing experience of being located inside our bodies. Here we report a perceptual illusion of body-swapping that addresses directly this issue. Manipulation of the visual perspective, in combination with the receipt of correlated multisensory information from the body was sufficient to trigger the illusion that another person's body or an artificial body was one's own. This effect was so strong that people could experience being in another person's body when facing their own body and shaking hands with it. Our results are of fundamental importance because they identify the perceptual processes that produce the feeling of ownership of one's body.

## Introduction

We all experience our body to be part of ourselves. The question of how this comes about has been discussed by philosophers and psychologists for centuries [1], [2], [3], [4]. Recent advances in experimental science have made it possible for cognitive neuroscientists to begin to investigate how we perceive our body as an object distinct from the external world [2], [5], [6], [7], [8]. Having the experience of being the owner of one's body is clearly adaptive, and its function probably relates to the problem of localising and correctly identifying oneself in the sensory environment [9], [10], a problem faced by all central nervous systems [11]. Consider a fight between two or more individuals. Survival depends on rapid identification and accurate localisation of one's own body. From neurology we know that these functions can break down as people with pathological conditions affecting frontal and parietal lobes can sometimes fail to recognise their limbs as belonging to themselves [12], [13], [14]. Similarly, damage to, or abnormal physiology of, frontal, parietal and temporal regions can be associated with feelings of being outside the body [15], [16]. Although these neurological observations suggest that certain brain regions might be responsible for generating the habitual experience of being located within one's body and of owning it, they tell us little about the underlying processes.

If we want to understand why our centre of awareness, or sense of ‘self’, is located inside our body, illusions of bodily self-perception could be invaluable. The study of illusions is a classical approach adopted in psychology to learn more about the basic processes that underlie normal perception. Indeed, some important initial insights into the mechanisms underlying self-perception of one's own body have been gained through illusions. One such is the so called ‘Rubber Hand Illusion’ where people have the experience that a prosthetic hand is actually their own hand [17]. In this illusion, synchronous touches applied to a rubber hand in full view of the participant, and the real hand, which is hidden behind a screen, produce the sensations that the touch originates from the rubber hand and a feeling of ownership of the artificial hand. This suggests that the temporal and spatial patterns of visual and somatosensory signals play an important role in how we come to experience that a limb is part of our own body [5], [7], [18], [19].

Another important factor in determining how we perceive our own body is the adoption of the first person perspective [6], [20]. When we look at ourselves directly, our limbs and body always present themselves in certain orientations because our eyes are fixed to our skull. By changing the visual perspective, it is possible to induce the feeling of being in a different place [21], [22], [23] or, even, illusory ‘out-of-body experiences’ where people seem to lose ownership of their own body when observing it from the point of view of another person (which we refer to as the third person perspective) [6].

On the basis of this previous knowledge, we hypothesized that it would be possible to induce illusions of owning an entire body other than one's own by the experimental manipulation of the visual perspective in conjunction with correlated visual and sensory signals being supplied to the respondent's body. Our experiments reveal that healthy volunteers can indeed experience other people's bodies, as well as artificial bodies, as being their own. This effect is so robust that, while experiencing being in another person's body, a participant can face his or her biological body and shake hands with it without breaking the illusion. The existence of this illusion (and the identification of the factors triggering it) represents a major advance because it informs us about the processes that make us feel that we own our body in its entirety.

## Results

### Ownership of a body other than one's own

#### Experiment # 1

**Figure 1:**
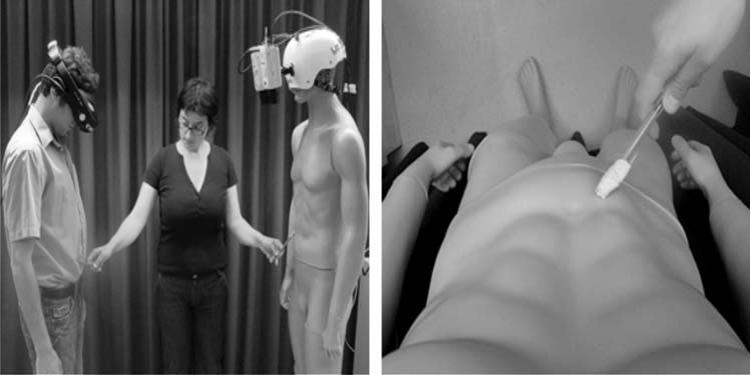
Set-up. Experimental set-up to induce illusory ownership of an artificial body (left panel). The participant could see the mannequin's body from the perspective of the mannequin's head (right panel).

The aim of the first experiment was to demonstrate that it is possible to elicit the illusion of ownership of an entire body. The experimental manipulation consisted of seeing a body other than oneself from the first person perspective whilst being subjected to synchronised visual and tactile stimulation. We used a life-sized mannequin, rather than another person's body, to exclude mismatches between small involuntary movements (e.g. breathing). To provide the first-person visual perspective of the other body, we developed the following set-up: Two CCTV cameras were positioned on a male mannequin such that each recorded events from the position corresponding to one of the mannequin's eyes. A set of head mounted displays (HMD) connected to the cameras was worn by the participants, and connected in such a way that the images from the left and right video cameras were presented on the left and right eye displays, respectively, providing a true stereoscopic image. Participants were asked to tilt their heads downwards as if looking down at their bodies. Thus, the participants saw the mannequin's body where they expected to see their own (Figure 1).

**Figure 2:**
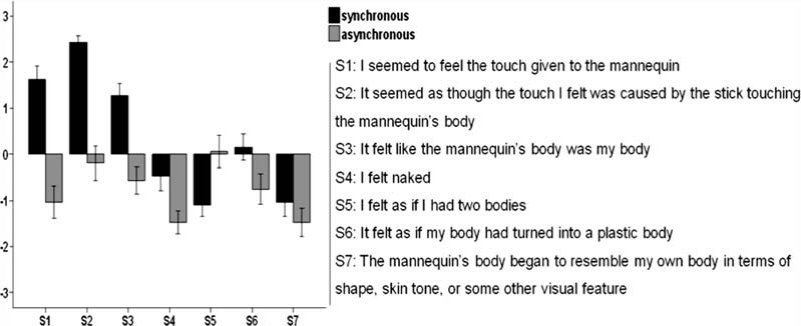
Questionnaire evidence for perceiving a mannequin's body as one's own. The questionnaire consisted of the seven statements (S1–S7). S1–S3 referred to the illusion and S4–S7 served as controls. Participants indicated their responses on a seven-step scale ranging from ‘agree strongly’ (+3) to ‘disagree strongly’ (−3). The high rating scores on the illusion statements that were observed only in the synchronous condition revealed that the participants experienced the illusion. The bars represent mean values and the error bars indicate standard errors. For details see Results and Methods.

We used a short rod to repetitively stroke the participant's abdomen, which was out of view, in synchrony with identical strokes being applied to the mannequin's abdomen in full view of the participant. As a control condition, we employed asynchronous touches to the real and artificial abdomens (carefully matching the total number and length of the strokes). After two minutes of stimulation, the participants were asked to complete a questionnaire on which they had to affirm or deny seven possible perceptual effects using a seven-point Likert scale. Three statements were designed to capture the illusory experience of being the artificial body, and the other four served as controls for suggestibility and task-compliance (Figure 2). From the completed questionnaires it was evident that the participants had felt the mannequin's body to be their own body, and that they sensed the touch of the rod directly on the mannequin's abdomen in the synchronous condition (p = .000, F(1, 223) = 125.434, ANOVA). No such illusory perceptions were reported in the asynchronous control condition (p = .703, F(1, 223) = 1.513, ANOVA). The responses to the three questions, which addressed the illusory perception of owning the new body, differed significantly between the two conditions (p = .000, F(1, 95) = 107.508, GLM for repeated measurements) (Figure 2).

### Physiological evidence for owning a new body

#### Experiment # 2

**Figure 3:**
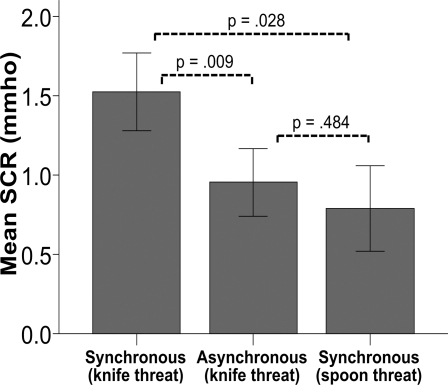
Physiological evidence for perceiving a mannequin's body as one's own. The mean skin conductance responses (SCRs) for 10 participants when the illusory body was “threatened” with either a knife or a spoon. The SCR is significantly greater in the illusion condition than in either of the control conditions (p = .009 and p = .028, two-tailed t-test). The response does not differ significantly between the two control conditions (p = .484, two-tailed t-test). Error bars denote standard errors.

To provide objective evidence for the illusion of owning a body, we threatened the mannequin and measured the evoked skin conductance response (SCR) as an objective measure of anxiety. This test has been used before to provide physiological evidence of body illusions [24], [25], and there is a direct relationship between the degree of anxiety evoked by threatening an artificial body part and the strength of illusory body ownership [25]. After a period of one minute of synchronous or asynchronous stimulation as described above, participants observed a knife ‘cutting’ the mannequin's abdomen. To control for a general effect of seeing an object approaching the body we also included a second control condition where we touched the mannequin's abdomen with a neutral object (a spoon of the same size as the knife) after one minute of synchronous and asynchronous visual and tactile stimulation, that is, after the same amount and kind of stimulation used in the “threatening” situation (see Methods). The key observation was a significantly greater SCR when we threatened the artificial body with the knife in the synchronous condition than in either one of the two control conditions (N = 10, p = .009, Z = −2.599, p = .028, Z = −2.191, two-tailed Wilcoxon Signed Ranks Test; see Figure 3). (This response was also significantly stronger than the low level control condition with the spoon threat after asynchronous stimulation; p = .017, Z = −2.395; not shown in Figure 3). There was no significant difference between the knife vs. spoon threat-evoked responses in the asynchronous condition (N = 10, p = .484, Z = −.700, two-tailed Wilcoxon Signed Ranks Test, the data are not shown). Thus, the participants' emotional systems responded as one would anticipate a person to respond were their own body being threatened.

#### Experiment # 3

**Figure 4:**
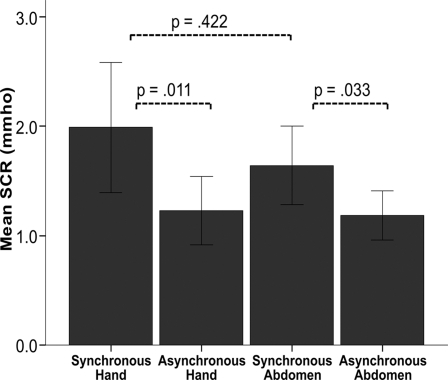
Generalisation of body ownership from the stimulated body part to the rest of the body. The mean skin conductance responses (SCRs) for 13 participants when the abdomen of the mannequin was “threatened” with a knife after a period of synchronous or asynchronous visuo-tactile stimulation of the hands or the abdomen (the four conditions on the x axis). The SCR is significantly greater in the synchronous (illusion) conditions than in the control ones regardless of whether the hands or the abdomen were stimulated (p = .011 and p = .033, two tailed Wilcoxon Signed Ranks Test). Mean values and standard errors are displayed.

In an additional control experiment we ruled out the possibility that the threat-evoked anxiety responses were limited to the particular body part that had been stimulated (i.e. the abdomen). To this end, we compared the magnitude of the SCR evoked by threats towards the abdomen after periods of synchronous or asynchronous visual and tactile stimulation of either the hands or the abdomen. The key observation was a significantly higher threat-evoked SCR after synchronous visuo-tactile stimulation of either the hands or the abdomen as compared to the asynchronous control conditions (N = 13, p = 0.011, Z = −2.511 and p = 0.033, Z = −2.132, two-tailed Wilcoxon Signed Ranks Test) (Figure 4). Thus, stimulation of one body part seems to produce ownership of the entire body being seen, i.e. the effect of ownership generalised to non-stimulated body parts. These findings together with the questionnaire data and spontaneous comments made after the experiments, conclusively demonstrated that people had the experience that the entire artificial body was their own body.

#### Experiment # 4

**Figure 5:**
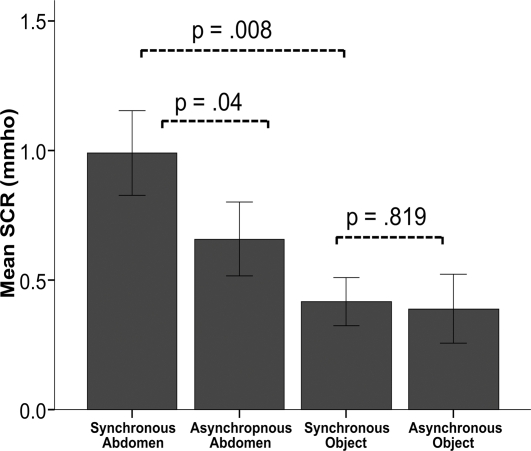
Only an object that looks like a human body can be owned. The figure displays the skin conductance responses (SCRs) from 12 participants when the mannequin and a rectangular object (a green box of the same size) were threatened in turn. The threat-evoked SCR was significantly greater when the mannequin was threatened in the synchronous condition than when the rectangular object was under threat after the same synchronous stimulation (p = .008, two-tailed t-test). A significant effect of synchronous vs. asynchronous stimulation was observed only when the mannequin was threatened (p = .04, two tailed t-test). Mean values and standard errors are displayed.

We also conducted an experiment to examine our prediction that the body would need to look like a human to be experienced as one's own. Pilot experiments suggested that the illusion did not work with objects that do not resemble a human body, such as boxes, chairs and tables. Thus we conducted an experiment where we applied threats to the mannequin or to a rectangular object of the same size, after a period of synchronous or asynchronous visuo-tactile stimulation to the respondent and the object. This experimental manipulation was also important because it allowed us to eliminate the potentially confounding factor that associated learning in the synchronous condition could have caused the differences in SRC responses in the previous experiments. The results of the data analysis revealed that the threat-evoked SCRs were significantly stronger in the synchronous condition with the mannequin than in the synchronous one with the rectangular object. (N = 12, p = .008, two-tailed t-test) (Figure 5). For a third time we reproduced a difference in the SCRs between synchronous and asynchronous stimulation of the humanoid body (N = 12, p = .04, two-tailed t-test), without any such difference being observed in the conditions with the rectangular object (p = .819, two-tailed t-test). Thus, people can only experience human-like bodies as part of themselves. In addition the differences in the SCR between synchronous and asynchronous conditions are highly specific for the illusion.

### Swapping body with another person

#### Experiment # 5

In a full-blown body-swap experience one would expect to be able to perceive being localized in another human's body during the performance of everyday actions. Furthermore, if this is a genuine perceptual illusion, it should be cognitively impregnable, and thus not break down even if one sees one's own body. The next experiment was designed to address these issues and to put the idea of illusory body-swaps to a hard test. We examined the counter-intuitive prediction that people should be able to swap bodies with each other and, quite literally, to shake hands with themselves while experiencing ownership of another person's body.

**Figure 6:**
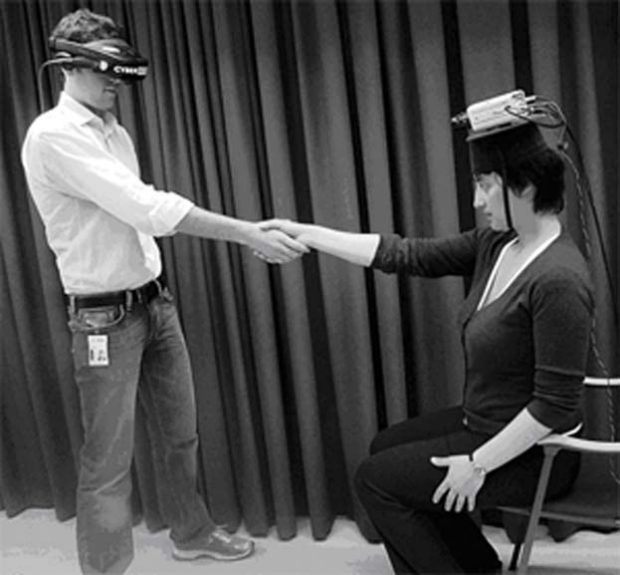
Experimental set-up to induce the ‘body swap illusion’.

In this experiment, the experimenter was wearing a specially designed helmet equipped with two CCTV cameras mounted in such a way that they presented the viewpoint of the experimenter (Figure 6). In turn, the participants stood directly opposite the experimenter, wearing the HMDs, which were connected to the CCTV cameras on the experimenter's head. Thus, the participants were facing the cameras. The participants were asked to stretch out their right arm and take hold of the experimenter's right hand, as if to shake it. This set-up allowed the participants to see their physical bodies from the shoulders to slightly above the knees. Hence, they could clearly recognize themselves and distinguish between their own arm and the arm of the experimenter. During the experiment, the participant and the experimenter were asked to repeatedly squeeze each other's hands for two minutes. In the illusion condition, the participant and the experimenter squeezed their hands in a synchronous manner, whereas in the control condition they squeezed each other's hands in an alternating rhythm, with the experimenter returning the squeeze in a semi-random manner. We tested the set-up in pilot experiments with ten participants (none of whom participated in the experiment reported here). Interviews conducted immediately after these initial experiments demonstrated that this set-up evoked a vivid illusion that the experimenter's arm was the participant's own arm and that the participants could sense their entire body just behind this arm. Most remarkably, the participants' sensations of the tactile and muscular stimulation elicited by the squeezing of the hands seemed to originate from the experimenter's hand, and not from their own clearly visible hand. In six other participants we also observed that this illusion worked well when the cameras were tilted downwards so that the participant could see the torso, legs and both arms of the experimenter's body during the manual interaction.

**Figure 7:**
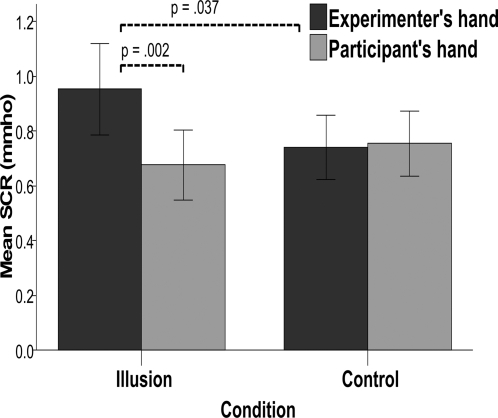
Objective evidence that people can experience swapping body with other people. Mean skin conductance responses (SCR) for twenty participants when either the experimenter's or the participant's hands were threatened during the illusion and the three control conditions (error bars represent standard errors). A significantly higher SRC was registered when the ‘new’ body (the experimenter's hand) was threatened with the knife in the illusion condition than when it was threatened in the control condition (p = 0.002, paired two-tailed Wilcoxon Signed Ranks Test) or when the physical ‘old’ body was threatened in the illusion condition (p = .037, paired two-tailed Wilcoxon Signed Ranks Test). The interaction between the main factors (Hand x Condition) was significant (p = .001, Two Way Repeated Measures ANOVA on standardized variables).

With the intention of obtaining objective and quantifiable data for this effect, we again used the procedure of threatening the bodies and measuring the SCR. We employed an experimental design where we occasionally threatened either the experimenter's hand or the participant's hand during either the synchronous or the asynchronous condition. As a threatening stimulus, we moved a knife just above the wrists as if cutting the hand. Identical plasters were placed on the wrists of the experimenter's and the participant's hands to make this procedure safe and not too scary (see Methods). We observed significantly stronger skin conductance responses when the knife was moved near the experimenter's wrist than when it was moved towards the participant's own hand in the synchronous condition (N = 20, p = .0002, Z = −3.099, two-tailed Wilcoxon Signed Ranks Test) (Figure 7). No such difference was observed during the asynchronous control condition, which did not elicit a vivid illusion (N = 20, p = 0.737, Z = −.336, two-tailed Wilcoxon Signed Ranks Test). The difference in SCR observed when threatening the experimenter's and participant's wrists in the two conditions was significant [interaction between the main factors Hands (Experimenter's vs. Participant's hand) vs. Condition (Synchronous vs. Asynchronous) was significant (p = 0.001, F(1, 19) = 17.083, Two Way Repeated Measures ANOVA on standardized variables]. Thus, the participants' emotional systems reacted more strongly when the new body was threatened than when their own body was under threat. This is a quite remarkable observation that speaks of the strength of the illusion. It is noteworthy that, after the experiment, several of the participants spontaneously remarked: “Your arm felt like it was my arm, and I was behind it”, “I felt that my real/own body was someone else” or “I was shaking hands with myself!” Finally, we registered a greater SCR when the experimenter's hand was threatened in the synchronous condition than in the asynchronous one (N = 20, p = 0.037, Z = −2.091, two-tailed Wilcoxon Signed Ranks Test). Thus, the SCR results cannot be explained in terms of a general emotional response to seeing the knife, nor can they be explained by differences in the distance between the knife and the cameras because these effects were all controlled for in our experimental design.

It is relevant to point out here that several of the participants also reported a weaker illusion in the asynchronous condition. Thus, even though they were able to recognize their own body through the headsets and visually detect the self-produced squeezing movements of their own hand, they were still influenced by seeing the experimenter's body from the first person perspective. This effect probably explains the lack of difference in the SCR observed when threatening the two hands in the asynchronous condition. Even though the mean rank of the observed SCR after threatening the participants' own hand was higher than that obtained by threat to the experimenter's hand in that condition (13.71 vs. 8.77), the effect was not statistically significant (N = 20,  = .737, Z = −.336, two-tailed Wilcoxon Signed Ranks Test).

### Body swap and gender

**Figure 8:**
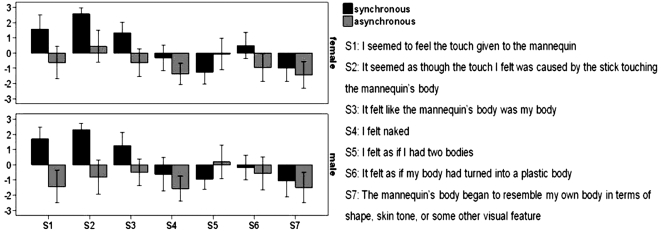
Questionnaire results for both sexes. The questionnaire consisted of the seven statements (S1–S7). There was no significant difference between the responses of female and male participants in the synchronous condition (p = .613, F(1,223) = .257, ANOVA). For details, see the Results and Methods.

The body-swap illusions worked well even though the mannequin or the other person looked different from the participant. In the first experiment there was no significant difference in rating scores between male and female subjects in the synchronous illusion condition, despite the fact that we only used a male mannequin (N = 32, p = .613, F(1,223) = .257, ANOVA) (Figure 8). Similarly, in the second experiment, male and female subjects alike were able to accept the arm of the female experimenter as their own. Further, we compared the threat-evoked skin conductance responses between males and females after threatening the new artificial body. To obtain sufficient numbers of males and females to enable a statistical comparison of the SCR, we pooled the data from the synchronous and asynchronous conditions where the stimulation was applied on the abdomen in experiments three and four. We found no significant difference in the illusion related SCR between males and females (p = .952, F = .004, Two Way Repeated Measures ANOVA). These observations suggest that gender identity, and differences in the precise shape of the bodies, are not important factors for perceiving a body as one's own.

## Discussion

The results of our experiments demonstrate that healthy volunteers can perceive another person's body, or an artificial humanoid body, to be their own. This works both when the participant does not move and when he or she is executing voluntary movements. The critical conditions for eliciting this perceptual illusion are: (i) a continuous match between visual and somatosensory information about the state of the body; (ii) the usage of a sufficiently humanoid body; and (iii) the adoption of a first person visual perspective of the body. Particularly strong evidence for this was the counter-intuitive demonstration that one can face one's physical body and shake hands with it whilst experiencing the illusion of being within another person's body. These findings are of fundamental importance because they identify the perceptual processes that make us feel that we own our entire body. Our results also provide a new method to move a person's perceived centre of awareness from one body to another, which could have important scientific, industrial and clinical applications (as will be discussed below).

Objective evidence for the illusion of owning an entire body was obtained by demonstrating that physical threats to the ‘new’ body elicited increased sweating (skin conductance responses) when people experienced the illusion. Similarly, threats to the ‘new body’ evoked greater skin conductance responses than threats to the ‘old’ physical one during the illusory body swapping. The SCR elicited by physical threats is a particularly good objective measure of body ownership. Emotional defence reactions, such as fear and autonomic arousal, have emerged in the course of evolution to enable one to protect one's own body from physical damage [26], [27]. The causal chain between threat-evoked SCR and the feeling of body ownership has been clarified in earlier work. There is a direct linear relationship between the strength of ownership of a hand and the degree of anxiety experienced when the hand is being subjected to physical threats [25]. This relationship is mirrored by the activity in multisensory areas, related to ownership, and areas in the emotional system, related to anxiety and pain anticipation [25]. The SCR is the peripheral correlate of activity in brain structures related to emotions, and is, therefore, greater when an owned rubber hand is physically injured than when it is not experienced as part of one's body [24]. Thus the differences in threat-evoked SCR that we observed in response to our experimental manipulations reflected the perceptual illusion of owning an entire body. The design of our experiments, employing multiple control conditions, ruled out potentially confounding factors such as attention to unexpected sensory events, the emotional salience of the stimuli presented, or associative learning (see Results for details).

The central relevance of the present findings is that they inform us about how we come to experience that we own our bodies and why we have an on-going feeling of being located inside them (sometimes called ‘embodiment’ [28]). In the present illusions, the visual, tactile, proprioceptive information and the predicted sensory feedback from these modalities during active movements were temporally and spatially congruent in an ego-centric reference frame centred on a new body. Thus, the matching of multisensory and motor signals from the first person perspective is sufficient to create a full sense of ownership of one's own entire body. This conclusion certainly contrasts with the traditional text-book wisdom which emphasises that body perception is a direct result of bottom-up processing of afferent signals from muscles, joints and skin.

Earlier work on body ownership has exclusively studied a single limb, the arm, using the traditional rubber hand illusion. Thus, until now, it was not known whether the principles underlying changes in ownership would generalise to other body parts, or to an entire body. Many multisensory brain areas seem to have particularly large representations of the hands and upper limbs [29], [30], and visual information is particularly important for guiding human hand actions [31], [32], [33], [34] and localising the arms in space [35]. The present data show that correlations of visual and tactile signals coming to a single body part (the abdomen or a hand) are sufficient to cause a feeling of ownership of the entire body (referred to as ‘whole-body ownership’). Thus the effect of correlated visual and tactile events on one limb generalises to non-stimulated body parts. This implies that visual and somatosensory signals from different body parts are analysed and interpreted together, i.e. that there are inter-dependences between the multisensory integration of different body parts. The central body representation is a ‘map of connected nodes’ where limbs and body segments form a continuous whole. It is this structure of the body representation coupled with the fact that the perceptual systems tend to produce single resolutions when resolving sensory conflicts [36], [37], [38] that makes whole-body illusions possible. The feeling of ownership of an entire body is , therefore, the result of a consistent pattern of spatially and temporally congruent multisensory signals from all body parts and the integration of this information in ego-centric reference frames centred on the various limbs and segments (that is, arm-centred, head-centred, etc).

Our data also directly demonstrate that visual information from the first person perspective is critical for the experience of owning a body. In our ‘body swap’ experiment (Experiment #5) there was a direct conflict between the perspective seen from the new body and the sight of the real body observed from a third person perspective. The synchronized movements of the two people squeezing each other's hands did not provide evidence in favour of either body being one's own, so the critical determinant for ownership in this set-up was the visual perspective. The first person perspective was clearly dominant since most participants experienced being in the new body. This dominance of the first person perspective probably explains why people did not show a very strong response when their actual body was threatened in the asynchronous condition. The incongruent tactile and muscular information in the asynchronous condition led to a decrease in the strength of the illusion, but could not completely abolish it, as reported by many participants. Importantly, the effect of varying the timing of the seen and the felt hand movements was significant only from the first-person perspective. The reason for this probably relates to the fact that central processing of multisensory signals from the body operates in egocentric reference frames [18], [30], [32], [39], [40], [41], [42], [43] which presupposes the first person perspective.

But why is bodily perception so malleable? The function of body ownership is probably related to the problem of localizing and correctly identifying the body in space. In this process, speed and accuracy are of utmost importance. According to recent statistical models of perception, the interpretation of multisensory signals could be sped up and made more precise by incorporating memory information (‘priors’) in the decision process [44], [45], [46]. In this framework, the body-swap illusions would arise as a result of the brain's tendency to rely heavily on a lifetime of experiences of seeing the world from the first person perspective with eyes that are fixed at a specific position on the skull, and the fact that the body typically produces certain patterns of sensory signals from the different modalities. Thus it is likely that the multisensory mechanisms involved in producing the present illusions are continuously engaged to refine the central estimate of the location of one's own body in natural situations. This implies that, in the experiments reported here, the spatial dimensions experienced as being occupied by one's own body, and the co-localisation of this sensed body and the physical body, are produced by this perceptual integration mechanism. This would explain why we have an on-going experience of being located inside our bodies.

The present illusions are consistent with the physiological and anatomical organisation of the multisensory brain. It is possible that bimodal and multimodal cells in premotor and posterior parietal areas could play important roles by mediating critical multisensory integration [7], [41], [42], [43], [47]. These areas are part of a system that controls actions and they contain many neurons that integrate visual, tactile and proprioceptive information in head and body-part centred reference frames [7], [41], [42], [43], [47]. Furthermore, these multisensory cells are sensitive to the temporal and spatial congruency of multisensory signals [47]. Thus, this neuronal system has the capacity to perform the binding of visual, tactile, proprioceptive and motor signals in ego-centric coordinate systems centred on the new body.

The sensory experience of one's own body from the first person perspective is different from recognising oneself in mirrors, TV screens or other image representations as observed from a third person perspective. When we recognise ourselves in a mirror, for example, we do not have the experience that we are actually in the mirror, or that there has been a change in ownership of our body. It is therefore likely that the present illusions involve different processes than those probed in earlier experiments on self-recognition in mirrors [48] or self-recognition of bodies [8], [49] and body parts observed from a third person perspective. A key difference probably is that, in the body-swap illusions, the visual, tactile and proprioceptive information is mapped directly onto the multisensory neuronal populations that represent one's own body in ego-centric coordinates.

The present findings could have groundbreaking industrial and clinical applications. Experiencing swapping bodies with other individuals could provide a valuable tool for research on body image disorders or self-identity in social psychology. Likewise, experiencing ‘becoming’ a humanoid robot in tele-robotics and feeling ownership of simulated bodies in virtual reality applications would probably enhance user control, realism, and the feeling of ‘presence’ [21], [22], [23], [50]. With respect to the tele-operator literature, it is interesting to note that there are many anecdotal reports of people feeling a robotic arm to be like their own when the robot arm is viewed from a first person perspective via cameras mounted on the robot and when the movements of the robot's arms reproduce the person's movements in real time [51]. The present paper provides the experimental data and a cognitive-neuroscience-informed model for explaining how illusory ownership of limbs and entire bodies might be evoked in tele-operator systems (see also Slater et al. [50]).

In conclusion, these experiments have demonstrated how remarkably easy it is to ‘move’ a human centre of awareness from one body to another. This speaks directly to the classical question of the relationship between human consciousness and the body, which has been discussed by philosophers, psychologists, and theologians for centuries [1], [3], [4], [11]. The continuous integration of multisensory and motor signals in ego-centric reference frames thus maintains the co-alignment between the experienced self and the physical body.

## Methods

### Participants

For each of the five experiments, we recruited separate groups of naïve healthy volunteers. In the first experiment, we tested thirty-two young adults (sixteen females, mean age 25±6 years). The second experiment involved ten individuals (two females, mean age 25±4 years), the third one consisted of a group of thirteen participants (eight females, mean age 27±6.5 years), and the fourth one was another group of twelve volunteers (four females, mean age 29±6 years). For the fifth experiment, twenty participants (13 females, mean age = 27±6.5 years) were recruited. All participants gave their written informed consent prior to participating in the relevant experiment. The studies were approved by the local Ethical Committee of Karolinska Institute.

### HMDs

In all experiments, participants wore a set of head-mounted displays, HMD, (Cybermind Visette Pro PAL, Cybermind Interactive, Maastricht, the Netherlands; Display Resolution = 640×480; true stereoscopic vision) with a wide field-of-view (diagonal field of view = 71.5°). These were connected to two synchronized colour CCTV cameras (Protos IV, Vista, Wokingham, Berkshire, UK) attached side-by-side to special helmets. The spacing between the cameras was adjusted for each participant to ensure that it matched the distance between their eyes (8–10 cm). The CCTV signals were relayed directly to the HMDs, without any software conversion, and thus were presented without noticeable delay. In experiments one, two, three, and four, the participants could clearly recognise the mannequin as a mannequin and in experiment five, they could see their own body and that of the other person.

### Physiological recordings

#### Recording equipment

The skin conductance responses were recorded with a Biopac System MP150 (Goleta, USA). Two electrodes were attached to the index and middle fingers of the participants' left hands using Signa electrode gel (Parker Laboratories, INC., New Jersey, USA). The data were registered with a Biopac System MP150 (100 samples per second) and processed with the Biopac software Acqknowledge for Windows ACK100W. The participant wore the electrodes for a few minutes before starting the recording. The parameters of the recording were as follows: The gain switch was set to 5 µmho/V and the CAL2 Scale Value was set to 5. The timing of the threat events was indicated in the raw data files during the recordings by the experimenter pressing a key.

#### Experimental design

In experiments 2 to 5 we used the following experimental designs:

*Experiment #2* consisted of three sessions, each of which consisted of four one minute long periods of synchronous or asynchronous stroking (each stroke was approximately 3 cm long; about 60 strokes were applied per minute). At the end of each period, the mannequin was threatened either with a knife or with a spoon. The order of the stimulation conditions and the type of object used as threat were randomized across sessions and participants [e.g. (Sk As Ak Ss) (As Ss Sk Ak) (As Sk Ak Ss), where S and A stand for synchronous or asynchronous stimulation and k and s stand for knife or spoon threat, respectively]. The data obtained from threatening the body with the spoon in the asynchronous condition was used as a low level control and is not displayed in the figures.

In *Experiment # 3* we used a similar protocol. It again consisted of three sessions, each of which consisted of four one minute long periods of synchronous or asynchronous stroking of the hands or of the abdomen. At the end of each period, the abdomen of the mannequin was threatened with a knife; the type of stimulation and its location (i.e. hands vs. abdomen) were randomized across sessions and participants [e.g. (Sh Aa Ah Sa) (Aa Sa Sh Ah) (Aa Sh Ah Sa), where S and A stand for synchronous or asynchronous stimulation and h and a represent the place where the stimulation was applied, i.e., to the hands or the abdomen, respectively].

*Experiment # 4* was conducted according to similar protocol to that used in the second and third experiments (three, four minute long sessions, with four conditions). The conditions were synchronous or asynchronous visual and tactile stimulation of the mannequin's body or of an object that did not look like a human body. This object was a dark green rectangular box of the same height and width as the mannequin's body. The touches on the object were applied at the same height as on the mannequin's body (thus at the same distance from the cameras) and the knife was moved along its short side in the same way it was moved along the mannequin's abdomen. During the short breaks between the sessions when the mannequin was replaced by the object, the goggles were switched off to prevent the participants seeing what change was taking place.

*Experiment # 5*, where the experimenter and the participants squeezed each other's hands, consisted of four sessions lasting two minutes apiece. During the whole experiment we played a metronome out loud at 40 beats per minute to assist in providing a steady and regular rhythm of the squeezing of the hand motion. In each session we threatened the real arm or the “owned” new arm twice. Two sessions corresponded to the illusion condition, with synchronous hand squeezing being conducted, and two sessions to the asynchronous condition. The two sessions used in the experiment were repeated twice in a pseudo-randomized order [(1, 2, 2, 1) or (2, 1, 1, 2)] to minimize the effect of presentation order. Within each session we randomized the order of which hand was threatened (either the experimenter's hand or the participant's hand). Further, the order of sessions and presentations were balanced across individuals. Within each session we threatened the hands once every 25 to 35 seconds, with the exact timing being varied to avoid anticipatory effects.

#### SCR: stimulation procedure and analysis

**Figure 9:**
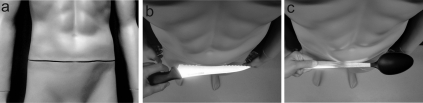
Illustration of the procedure to simulate cutting the mannequin with either a knife or a spoon in Experiment #2.

Each time that the knife or spoon was slid along the mannequin's abdomen (experiments 2, 3 and 4) or the knife moved above the wrists (experiment 5) took approximately 3 seconds. The motion was performed so that the knife and the spoon were always moved along the horizontal axis from left to right in the field of view of the HMDs. During the movement the object was inserted slightly into the mannequin's abdomen in a small gap between the upper and lower parts of the mannequin's body. To make this possible we placed two circular sticky patches (0.5 cm high, 1 cm diameter) between the torso and the lower part of the body of the mannequin, thereby, creating a cleft in the lower part of the abdomen of the mannequin that was not visible from the perspective of the cameras (Figure 9a). The objects used to provide the threat were moved along the abdomen in such a way that it looked as if the object was ‘cutting’ into the dummy's body from the perspective of the cameras (Figure 9b/c). In the fourth experiment the knife was run in full contact with the rectangular object, but we could not induce the visual effect of cutting into it because of its flat surface. For this particular experiment we adjusted the way that the knife threat was applied to the mannequin so that the knife was moved along touching the dummy's body, but without appearing to cut into it. Great care was taken to move the knife or the spoon in exactly the same way from trial to trial. The SCR was identified as the peak in the conductance that occurs up to 5 seconds after the onset of the threat stimuli. The amplitude of the increase in conductance was measured as the difference between the maximal and minimal value of the response identified in this time-window. We calculated the average of the all responses including the trials where no response was apparent, thus, analysing the magnitude of the SRC [52]. Participants who did not show a reliable threat-evoked SCR (‘null responders’), i.e. had zero responses in more than two-thirds of the trials, were excluded from the analysis.

#### Statistical analysis

The data obtained from the five different experiments were tested using the Kolmogorov-Smirnov test to determine whether they fitted the requirements for a normal distribution. Only the data from the first and the fourth experiments passed the normality test, therefore we used parametric statistical tests to process the data obtained in those two experiments and the data from the other three experiments were tested with non-parametric statistical tests. In all statistical tests, we set alpha to 5%.
